# Age and attitude: How longevity influences cognitive biases in honeybee workers

**DOI:** 10.1098/rspb.2025.1696

**Published:** 2025-10-22

**Authors:** Karolina Kuszewska, Aleksandra Żmuda, Anna Maria Gajda

**Affiliations:** ^1^ Department of Zoology and Animal Welfare, Faculty of Animal Science, University of Agriculture in Krakow, Kraków, Poland; ^2^ Doctoral School of Exact and Natural Sciences, Jagiellonian University, Kraków, Lesser Poland Voivodeship, Poland; ^3^ Institute of Botany, Faculty of Biology, Jagiellonian University, Kraków, Lesser Poland Voivodeship, Poland; ^4^ Institute of Veterinary Medicine, Warsaw University of Life Sciences, Warsaw, Masovian Voivodeship, Poland

**Keywords:** honeybee, judgement bias, expected longevity, rebel workers, *Nosema* infection, injury, anaesthesia, go/no-go task

## Abstract

This study examines how life expectancy influences cognitive biases in honeybee workers (*Apis mellifera*) and their emotional states affecting decision-making. Recent research indicates that emotions impact behaviour and cognition in various species, including invertebrates. We conducted three experiments to manipulate honeybee lifespan and assess its effects on judgement biases using a classic judgement bias paradigm. In the first experiment, we shortened the lifespan of bees through CO_2_ anaesthesia or thoracic puncture. The second experiment involved feeding workers *Nosema* sp. spores to induce infection and evaluate its impact on survival and judgement. The final experiment focused on ‘rebel workers’, who have naturally longer life expectancies. We conditioned bees to associate specific odours with rewards and punishments, then measured their responses to ambiguous stimuli. Results showed that bees subjected to life-shortening treatments exhibited more optimistic cognitive biases, as indicated by an increased likelihood of extending their proboscis to ambiguous odours. Conversely, rebel workers with longer lifespans displayed more pessimistic biases, indicating a cautious foraging approach. Overall, our findings suggest that honeybee decision-making is closely tied to their longevity, with shorter-lived individuals adopting riskier strategies compared to their longer-lived counterparts.

## 1. Introduction

The study of emotion in animals is still controversial and of immense societal concern [1]. Animals cannot tell us how they feel, and for this reason, we need to measure and evaluate inferred animal emotion using physiological, cognitive and behavioural metrics [2]. Emotions can influence many aspects of human as well as animal life, including attention, perception, memory, expectation and decision-making. In the past 10 years, studies have shown the existence of ‘primitive emotions’ (or affective states) in different groups of animals, such as fish [3,4], rats [5], pigs [6], sheep [7], goats [8] and dogs [9]. The existence of affective states has also recently been extended to invertebrates [2,10].

To evaluate affective states in invertebrates, researchers commonly utilize the classic judgement bias paradigm. In this approach, animals are trained to associate stimulus A with a reward (CS+) and stimulus B with the absence of reward or a mild punishment (CS−). Subsequently, they are tested with ambiguous stimuli that fall between A and B. A positive emotion-like state is inferred if animals respond to the intermediate stimulus as if it were equivalent to A, while a negative emotion-like state is suggested if they reject the intermediate stimulus, treating it as similar to B. Studies using this protocol have shown that honeybees exposed to simulated predatory attacks (e.g. being shaken in a tube) exhibit subsequent ‘pessimistic’ cognitive biases in decision-making [11]. Similarly, crayfish subjected to electric shocks avoid illuminated arms of an aquatic plus-shaped maze, a behaviour interpreted as indicating an ‘anxiety-like state’ [12]. Variations in cognitive judgement bias are often interpreted as reflections of underlying affective or emotion-like states, consistent with the original conceptualization of the test [5,13,14], although some researchers propose alternative explanations [2] and argue that interpreting these findings as motivational drives, rather than emotional states, could be a less biased and more cautious approach. The question of whether insects have emotional states or their equivalents is still widely debated [2,15]. However, conditioned judgement bias responses in invertebrates offer a way to observe valence-based processing behaviourally. We use a broad definition of ‘affect’ that encompasses all processes related to valence (‘pleasantness’ or ‘unpleasantness’), including emotions, moods and their potential physiological or behavioural expressions, regardless of whether they involve conscious awareness. This approach enables comparisons across different species without necessarily assuming that conscious experience is involved [16].

Most previous studies have investigated how the cognitive judgement bias of animals changes when exposed to positive or negative stimuli immediately before testing. However, none have examined how cognitive judgement bias change among groups of animals with differing life histories, which can influence physiology, lifespan, emotional state, and motivation for various activities. A key parameter associated with life history is life expectancy, which indicates the average duration an animal is likely to live. Life expectancy is not solely determined by an individual’s age, but also by life history factors such as exposure to infections, injuries and environmental influences during development. In social insects like bees, wasps and ants, life expectancy directly impacts the division of labour within the colony [17]. Research has shown that ants [18] and honeybees [19,20] with experimentally shortened life expectancies (due to injury, poisoning or parasitic infection) transition more quickly from safe inside-nest tasks to riskier outside-foraging tasks. Additionally, a study on behavioural reversion in honeybees revealed that foragers with shortened life expectancies were less likely to revert to safer, inside-nest tasks compared to same-age foragers in control groups [21]. Further research also indicated that reduced life expectancy not only affects the onset of foraging but also increases the likelihood of engaging in riskier tasks. Foraging ants with shortened life expectancies tended to forage at greater distances from the nest, in higher temperatures and in the presence of competitors more often than those in control groups [22]. Similarly, honeybee foragers with shortened life expectancies preferred collecting nectar over water, foraged during inclement weather rather than favourable conditions [17], and more frequently appropriated provisions stored in foreign nests compared to controls [23].

Life expectancy can also influence various aspects of worker behaviour related to interactions and communication among colony members. Numerous studies have shown that injured or infected honeybee workers tend to avoid brood care and limit contact with adult nestmates [24–27]. Similarly, old and moribund ant workers often do not call for help when they fall into traps [28]. These observations are consistent with previous findings regarding the social withdrawal of moribund workers, which can benefit colonies by reducing the potential spread of disease among individuals [24,28].

In this study, we aim to investigate how differences in life history, as measured by life expectancy, influence the affective states of honeybees (*Apis mellifera*) and their judgement biases. To achieve this, we conducted three experiments. In the first, we shortened the life expectancy of the workers by anaesthetizing them with CO_2_ or injuring them by puncturing their thorax. In the second experiment, we again shortened life expectancy but this time by infecting the workers with *Nosema* sp. spores. In the third experiment, we used workers that are naturally characterized by longer life expectancies—rebel workers. For all experiments, a portion of the workers was used to assess lifespan in a laboratory cage setting, while the remaining workers were employed in judgement bias and control tests. We measured judgement bias using the judgement bias paradigm in honeybees [29,30]. Initially, the bees were trained to associate two stimuli: the first with rewards (CS+) and the second with punishment (CS–). Subsequently, we tested their judgement bias by evaluating how they classified novel stimuli that had sensory properties intermediate between the two previously trained stimuli.

## 2. Methods

The research was conducted between May and June of 2018 and 2023 at the experimental apiary of the Institute of Environmental Sciences (Jagiellonian University, Krakow, southern Poland) and the University of Agriculture in Krakow (Krakow, southern Poland). A total of 14 unrelated queenright honeybee colonies of *Apis mellifera carnica* were studied across three experiments, each consisting of 20 000 to 40 000 worker bees.

### (a) General procedure

**Figure 1.**
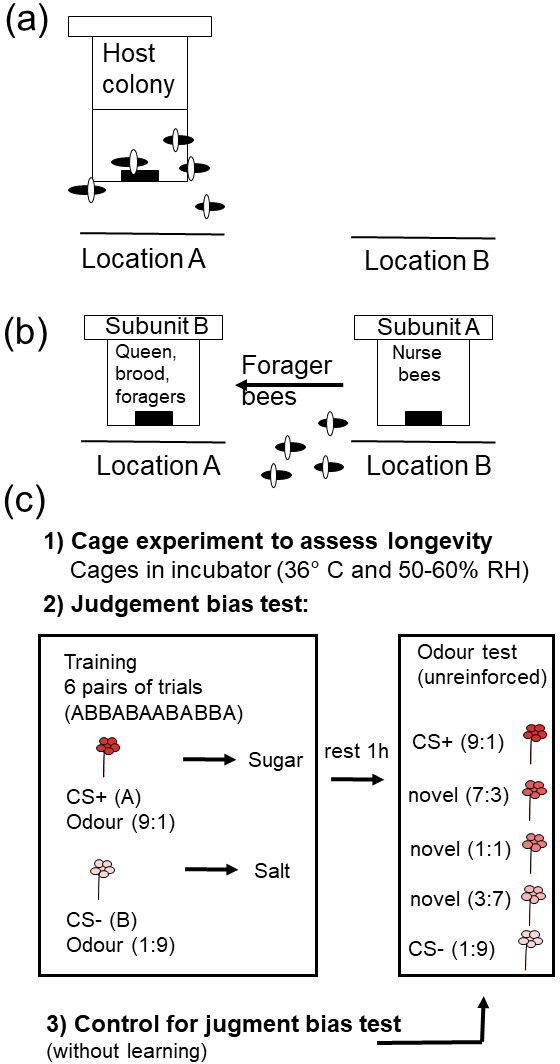
Schematic of the experiment. (a) Each of the six original host colonies at location 1. (b) All host colonies were split into two parts to separate foragers from other bees. Subunit A, consisting of the queen, all adult workers, and the combs containing honey, pollen and brood, was relocated several metres from the original hive site (location 1) to a new location (location 2). Subunit B, consisting of frames with honey and pollen and one frame with open brood, remained at the original hive site (location 1). As a result, foragers that left subunit A to collect food returned to subunit B. (c) Captured foragers from experiment 3 were randomly divided into three groups in experiment 1 (untreated control, anaesthetized with 99.9% CO_2_ for 30 min, and injured by puncturing the last segment of the thorax), and two groups in experiment 2 (untreated control and infected with *Nosema* spores) and experiment 3 (normal control workers and rebel workers). The workers from all groups were then used in three different tests. The first portion of the workers was used in a cage experiment to assess the longevity of bees from different treatments in the incubator. The second portion was used for the judgement bias test. The CS+ odour in half of the colonies was a mixture of nine parts 1-hexanol to one part 2-octanone, while the CS− was a combination of one part 1-hexanol and nine parts 2-octanone. In the other half of the colonies, the CS+ was a mixture of nine parts 2-octanone and one part 1-hexanol, while the CS− was composed of one part 2-octanone and nine parts 1-hexanol. After conditioning, bees were tested for proboscis extension response (PER) using each CS and three novel intermediate ratios of the same two odours. The order of the test odours was randomized across individuals, and all tests were unreinforced. The third portion of workers served as the control test for spontaneous responses. This part of the study was identical to the judgement bias test; however, the workers were not trained to recognize the two signals beforehand.

In each experiment, foraging workers were separated from non-foraging bees according to the protocol established by Kuszewska & Woyciechowski [21]. The honeybee colony was divided into two subunits (A and B) at 09.00, before the active foraging period began. Subunit A, which contained all worker bees along with frames of brood and food, was relocated several metres away from the original colony site. Subunit B, consisting of nine frames of food and one open brood frame, remained in its original location, with its entrance aligned with that of the native hive (figure 1a,b). This separation ensured that only nurse bees stayed in subunit A, as foraging bees returned to subunit B after foraging. In the afternoon (between 13.00 and 16.00), forager bees from subunit B were collected for the experiments.

### (b) Experiment 1: injury and CO_2_ lifespan reduction

In experiment 1, conducted with six unrelated honeybee colonies, the collected foragers were randomly divided into three groups: (i) untreated control, (ii) anaesthetized using 99.9% CO_2_ for 30 min and (iii) injured by puncturing the last segment of the thorax with a needle (0.35 mm diameter, depth to the first drop of haemolymph). Each group underwent three different tests (figure 1c): (i) cage experiment to assess longevity, (ii) judgement bias test and (iii) control test for spontaneous responses.

### (c) Experiment 2: *Nosema* infection lifespan reduction

For experiment 2, four unrelated honeybee colonies were used. The collected foragers were randomly divided into two groups: (i) untreated control and (ii) *Nosema* sp. spore-fed workers (infected group). Workers from both groups were individually fed a 10 µl solution of water and sugar (50% concentration), with the solution for the infected group additionally containing 1.75 × 10^4^ *Nosema* spores. After one week in a cage to allow infection development, the workers were divided into three parts for testing, similarly to experiment 1. Following the cognitive experiments, the bees were frozen and dissected to count the *Nosema* spores in their intestines. For this analysis, the digestive tract (excluding the crop) of each bee was homogenized in 300 μl of distilled water. *Nosema* spores were counted using a Bürker haemocytometer in a total solution volume of 1.25 × 10^−2^ μl. If the number of spores counted per sample was fewer than 10, the total solution volume for that sample was increased to 8 × 10^−2^ μl. The total number of spores per bee was then calculated using the following formula: number of spores per bee = (number of spores per sample × 300 μl) ÷ total solution volume of the sample [23].

### (d) Experiment 3: rebel workers life extension

In experiment 3, also involving four unrelated honeybee colonies, two groups of workers were reared: (i) normal workers with a typical life expectancy and (ii) rebel workers with an extended life expectancy (as per Kuszewska *et al*. [31]). Rebel workers develop under queenless conditions. They are distinguished by their reproductive investment: they have more ovarioles in their ovaries, more developed mandibular glands and underdeveloped hypopharyngeal glands [32]. To rear these groups, the queen was confined to two experimental frames to produce uniformly aged eggs. The colony was divided into queenright and queenless subunits, merged after all worker cells in the experimental frames were sealed [32]. Before the emergence of adult workers, the experimental frames were placed in an incubator (34°C, 90% RH). Workers were marked and returned to their colonies. When both groups were 15 days old, foragers were collected similarly to experiments 1 and 2 and used in three different tests. Following the cognitive experiments, 10 normal and rebel bees were collected, immediately frozen and then dissected to determine the total number of ovarioles in both ovaries of each worker. This ensured that we had two distinct groups of individuals [32,33].

### (e) Determination of worker lifespan in cage experiment

The workers were housed in wood-frame cages (13 × 9 × 5 cm) with glass and steel mesh sides, and were provided with a small piece of bee comb. In experiment 1, three cages were prepared for control, anaesthetized and injured workers. Experiment 2 featured two cages for control and infected workers, while experiment 3 included two cages for normal and rebel workers, all arranged for each experimental colony. The number of workers in each cage across all experiments was standardized at 55 individuals. However, due to unrelated injuries or manipulations resulting in death, the count was adjusted to 50 individuals for data consistency by removing the surplus bees the following day. Lifespan estimation began the next day. The cages were incubated at 36°C with 50–60% relative humidity, each containing a 50% sucrose solution and water ad libitum. The cages were checked daily, and deceased bees were counted and removed.

### (f) Judgement bias test

The workers were placed in a wood-frame cage (13 × 9 × 5 cm) for only 24 h to allow them to rest after the longevity manipulation. An exception were the bees from experiment 3, whose lifespan was determined during the larval stage. However, to ensure all experiments were methodologically consistent, these bees also stayed overnight (24 h) in the cage before the judgement bias test procedures began. The following day, after 24 h of rest, the bees were individually harnessed in a plastic conditioning station and secured to ensure immobility for conducting the proboscis extension reflex (PER) [34,35] learning procedure. After this, the subjects were fed 10 µl of a 50% sucrose solution within 30 min of restraint and were then kept in the dark for 2 h to serve as a rest phase.

Subsequently, the bees were conditioned according to methods similar to those described by Bateson *et al.* [11], and they were assigned to the judgement bias task category known as the go/no-go paradigm [36]. The conditioning involved presenting two odours, each paired with a distinct outcome, in a pseudorandom sequence (half ABBABAABABBA and half BAABABBABAAB, where A = CS+ and B = CS–) with an intertrial interval of 10 min for a total of 12 trials, using an established protocol for conditioned proboscis extension [35]. The odours 1-hexanol and 2-octanone (99.8% purity, Sigma-Aldrich) were used as the conditioned stimuli, as these volatile compounds have been employed in previous studies of honeybee olfactory learning [11,37]. The odours were combined as a binary mixture and used as conditioned stimuli in the following proportions: the 9 : 1 odour mixture (1-hexanol : 2-octanone or 2-octanone : 1-hexanol depending on the bees’ colony) served as a reward food solution (CS+, 50% sucrose), while the 1 : 9 odour mixture acted as a punishment (CS−, 30% saline NaCl). This selection of odour mixtures for the learning test was made to ensure that bees experienced different concentrations of these chemicals, consistent with those used in the cognitive biases test, thus providing them with relevant exposure during the conditioning phase.

While previous research has demonstrated that both mixtures are learnt equally well [38], the conditioning mixtures varied for bees from different colonies. Bees from experiment 1 (colonies 1, 3 and 5) as well as those from experiments 2 and 3 (colonies 1 and 3) were conditioned with a 1-hexanol : 2-octanone mixture (9 : 1) as the reward and (1 : 9) as the punishment. In contrast, bees from experiment 1 (colonies 2, 4 and 6) and experiments 2 and 3 (colonies 2 and 4) were conditioned with a 2-octanone : 1-hexanol mixture (9 : 1) as the reward and (1 : 9) as the punishment. Next, the bees had a brief rest period of 1 h in the dark before the cognitive biases test.

In the judgement bias test, the bees were exposed to a binary mixture of 1-hexanol and 2-octanone, incorporating additional intermediate proportions (3 : 7, 1 : 1 and 7 : 3) along with those used in the conditioning phase (9 : 1 and 1 : 9; figure 1c) [11]. Each bee was tested with all five stimuli without reinforcement, and the order of odour presentation was randomized across subjects. In experiment 1, bees were tested in groups of six (2 control, 2 injured and 2 anaesthetized), while in experiments 2 and 3, groups consisted of four bees (2 control and 2 infected or 2 control and 2 rebel), respectively. This grouping was done to mitigate potential confounding variables such as time or day. The number of bees at the end of each test varied across experiments, colonies and experimental groups. The specific numbers of bees used in each experiment are summarized in table 1.

**Table 1.** The number of bees used in the judgement bias test in each of the three experiments.

experiment 1						
	**colony 1**	**colony 2**	**colony 3**	**colony 4**	**colony 5**	**colony 6**
control	22	25	22	20	20	21
injury	20	26	20	20	24	21
CO_2_	20	22	21	22	23	20

experiment 2				
	**colony 1**	**colony 2**	**colony 3**	**colony 4**
control	42	42	44	50
infected	45	43	49	47

experiment 3				
	**colony 1**	**colony 2**	**colony 3**	**colony 4**
control	47	30	47	24
rebel workers	42	45	41	45

### (g) Control test for spontaneous responses

The third group of workers (10 from each experiment, colony and experimental group—180 in total for experiment 1, 80 for experiment 2 and 80 for experiment 3) was utilized in the control test of the judgement bias. Similar to the bees from the second group, these bees were held separately in a plastic conditioning station, fed 10 µl of a 50% sucrose solution within 30 min after restraint, and then kept in the dark for 2 h to serve as a rest phase. Following this rest period, these individuals were exposed directly to five different proportions of a binary mixture of 1-hexanol and 2-octanone (1 : 9, 3 : 7, 1 : 1, 7 : 3 and 9 : 1) without undergoing any conditioning learning phase. The order of odour presentation was randomized across subjects, similar to the cognitive biases test. This procedure verified whether the bees displayed a spontaneous proboscis extension reflex (PER) in response to the presented mix of odours.

### (h) Statistical analysis

Differences in survival among worker bees in our three experiments were analysed using the Kaplan–Meier survival test. In each experiment, the longevity of bees from different colonies and groups was compared. If significant differences were observed between the experimental groups, a log-rank test was performed to compare the two groups directly with Bonferroni correction.

We estimated differences in judgement bias among individuals using general linear models (GLMMs) with repeated measures, treating the bees' responses to different scent concentrations (9 : 1, 7 : 3, 1 : 1, 3 : 7 and 1 : 9) as dependent variables. The independent variables included worker type, modelled as a fixed factor (in experiment 1: control, injured and anaesthetized; in experiment 2: infected and uninfected; and in experiment 3: normal and rebel workers) and colony, included as a random factor. If the analyses indicated that the random factor was not significant, data from different colonies were combined. When the fixed factor was found to be significant, a post-hoc Tukey test was performed for different sample sizes in the first experiment (this primarily pertains to experiment 1, which involved three groups of tested workers: control, injured and anaesthetized workers.).

Additionally, in experiment 2, we examined whether workers from the control and infected groups (*Nosema*-fed) differed in their infection levels. We conducted this analysis because the forager bees collected may also be infected independently of our procedures. To test this, we used the non-parametric Kruskal–Wallis test and compared worker groups originating from different colonies and different experimental treatments (control and infected, totalling eight groups).

In experiment 3, we also assessed whether workers from the two different groups—normal and rebel workers—differed in the number of ovariole in each group and colony. For this comparison, we utilized a generalized linear model (GLM) with a poisson distribution. In software Statistica 13 this is referred to as a generalized linear/nonlinear model (GLZ). All calculations were performed using Statistica 13.3.

## 3. Results

### (a) Experiment 1

**Figure 2.**
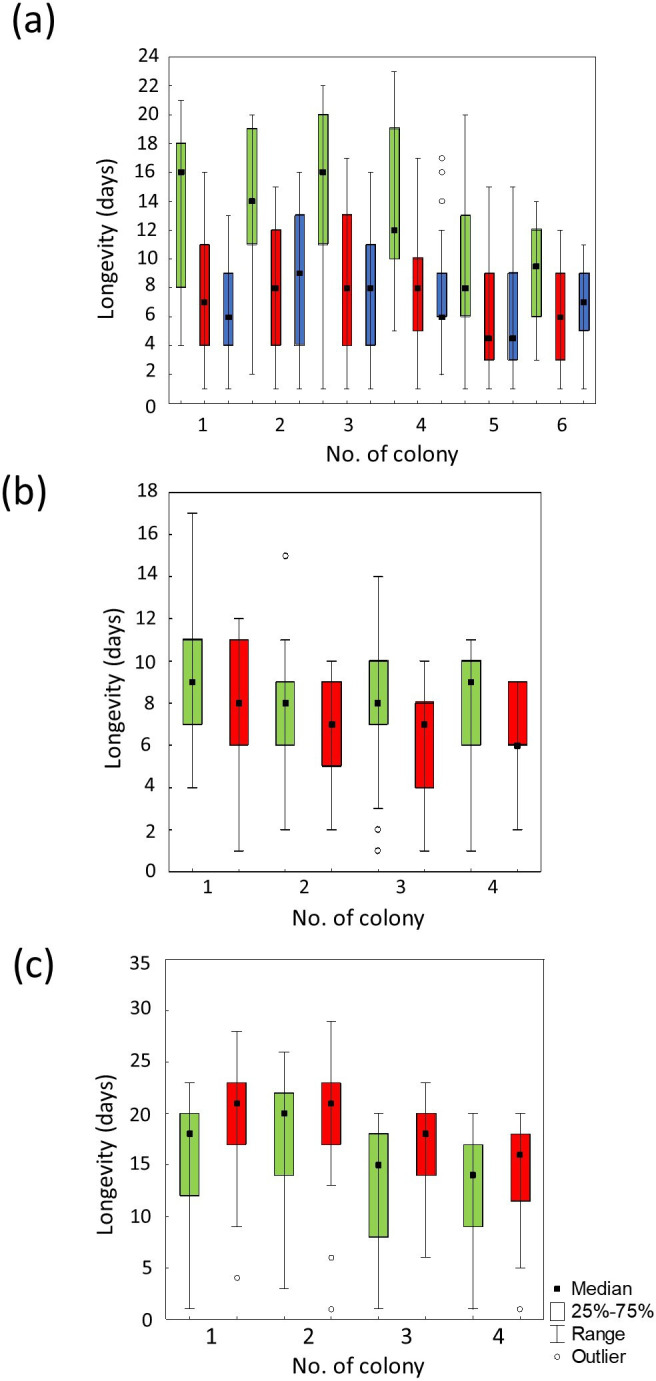
Longevity (median, quartiles, minimum and maximum) of the three experiments. (a) Experiment 1: control (green), injured (red) and CO_2_-anaesthetized (blue) workers in the cage experiment. (b) Experiment 2: control (green) and infected (red). (c) Experiment 3: control (green) and rebel (red). In each experiment, within each group and family, 50 individuals were tested for lifespan.

The lifespan of bees in the cages differed between individuals from different colonies (Kaplan–Meier survival analysis *χ*² = 60.83, d.f. = 5, *p* < 0.001; figure 2) and groups (*χ*² = 171.88, d.f. = 2, *p* < 0.001). The log-rank multiple comparison (with Bonferroni correction with *p* = 0.0167) showed that the control group differed from injured (log-rank, *Z* = −10.96; *p* < 0.001) and anaesthesia groups (log-rank, *Z* = −11.28; *p* < 0.001) while these two treated groups did not differ from each other (log-rank = −0.70; *p* = 0.482).

**Figure 3.**
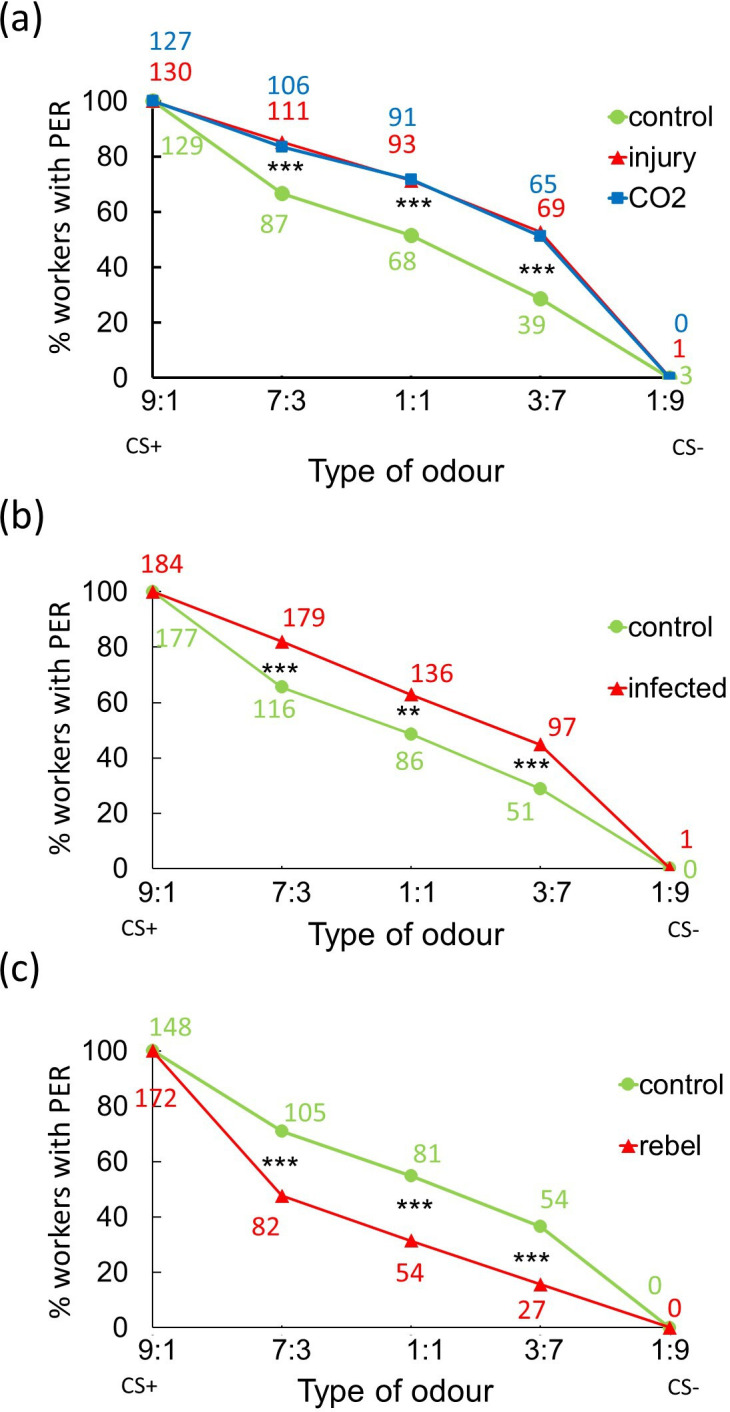
Results of the judgement bias test. (a) Experiment 1: percentage of workers from three groups (control, injured and CO_2_-anaesthetized) showing proboscis extension response (PER) to various odours (pooled data from all colonies). (b) Experiment 2: percentage of workers from two groups (control and infected) with PER reactions to different odours (pooled data from all colonies). (c) Experiment 3: percentage of workers from two groups (control and rebel) demonstrating PER to various odours (pooled data from all colonies). Asterisks indicate statistically significant differences between groups: *p < 0.05; **p < 0.01; ***p < 0.001. The coloured numbers represent the number of bees in each group exhibiting the PER.

The results of comparing PER reactions to different odours in the judgement bias test among individuals indicated that there were no significant differences between bees from different colonies (general linear mixed model with repeated measurement: d.f. = 5; *F* = 0.534; *p* = 0.751), and therefore, data from different colonies were combined. The analysis comparing PER reactions among workers from the control, injured and treated groups exposed to CO_₂_ in a binary mixture of 1-hexanol and 2-octanone showed no differences between these groups when exposed to the odours used during the conditioning phase (9 : 1—d.f. = 2; *F* < 0.001; *p* = 0.999; 1 : 9—d.f. = 2; *F* = 1.758; *p* = 0.174; figure 3a). However, there were significant differences between these groups in the intermediate odour proportions (7 : 3—d.f. = 2; *F* = 7.499; *p* < 0.001; 1 : 1—d.f. = 2; *F* = 6.841; *p* = 0.001; 3 : 7—d.f. = 2; *F* = 8.633; *p* < 0.001; figure 3a). More specific analyses revealed that control workers were less likely to extend their mouthparts in response to the three novel odours compared to injured and CO_₂_-anaesthetized workers across all odour proportions (Tukey test for different *N* : 7 : 3—control versus injured: *p* = 0.006; control versus CO_₂_: *p* = 0.030; 1 : 1—control versus injured: *p* = 0.002; control versus CO_₂_; *p* = 0.003; 3 : 7—control versus injured: *p* < 0.001; control versus CO_₂_, *p* < 0.003; figure 3a). However, there were no significant differences in proboscis extension between the two groups with shortened life expectancies across odour proportions (7 : 3*—p* = 1.000; 1 : 1*—p* = 1.000; 3 : 7*—p* = 1.000; figure 3a).

The results of the control test for spontaneous responses showed that only four workers of the 60 workers tested in each group (colonies and treatment) reacted in the control and injury groups, whereas only one individual reacted in the anaesthetized group.

### (b) Experiment 2

**Figure 4.**
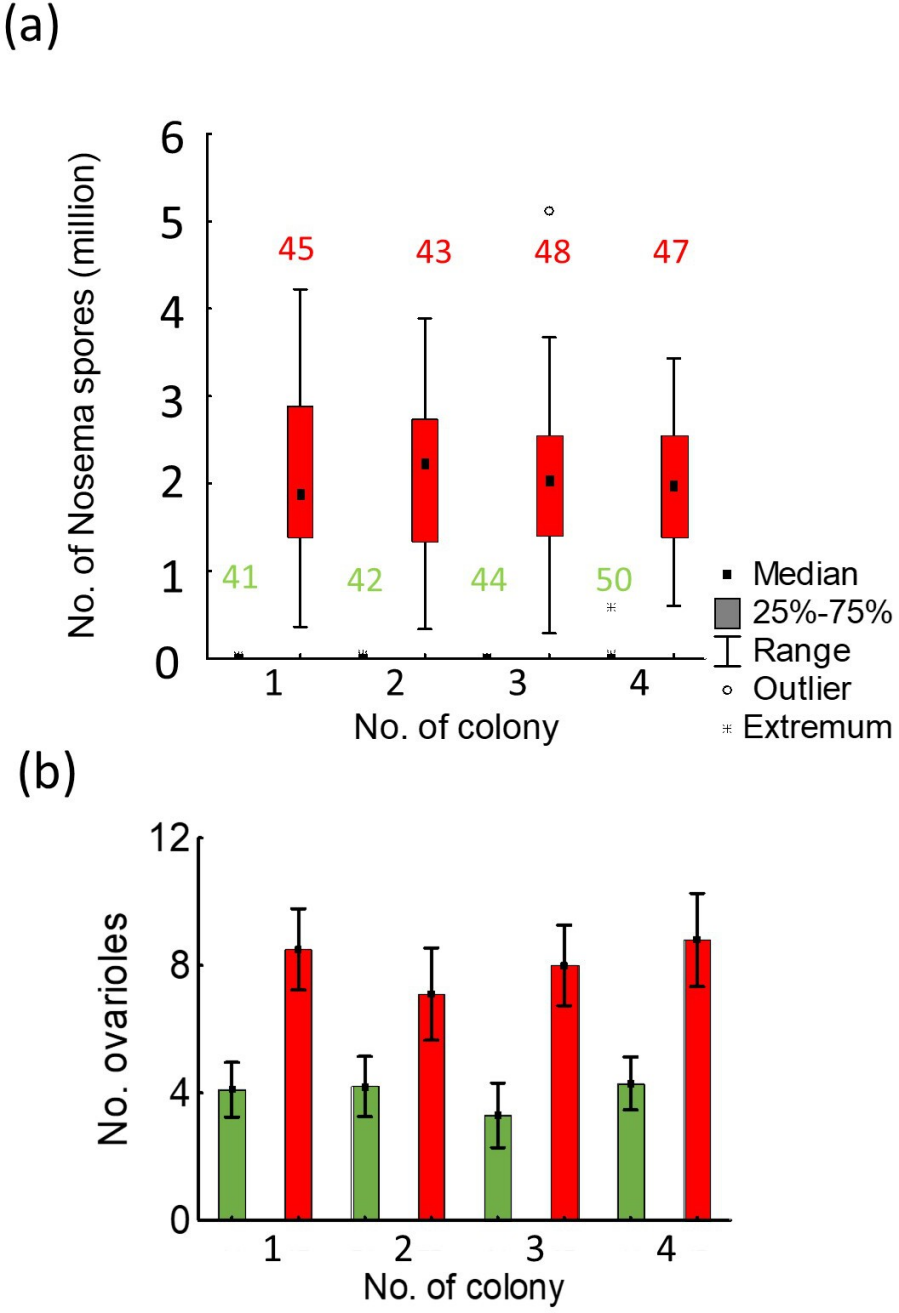
Analysis of (a) infection levels in experiment 2 and (b) ovariole counts in experiment 3 among worker bees. (a) Experiment 2—*Nosema* sp. infection: control workers (green) and infected workers (red). The numbers above the bars indicate the number of bees used to estimate *Nosema* sp. infection. (b) Experiment 3—number of ovarioles in control workers (green) and rebel workers (red). In each group and family, 10 individuals were tested for their ovariole number.

At the beginning, we checked whether the two groups differed in the number of *Nosema* sp. spores. The results indicated that bees from the control group typically had no spores in their digestive tracts, with only a small number of individuals infected (the number of individuals with *Nosema* spores: colony 1—4 bees; colony 2—4 bees; colony 3—5 bees and colony 4—0 bees). In contrast, all bees in the groups fed with *Nosema* spores were infected. The median tests revealed significant differences in the number of *Nosema* spores between the control and infected workers (*χ*^2^ = 340.595, d.f. = 7, *p* < 0.001; figure 4a,4). Furthermore, multiple Kruskal–Wallis comparisons showed that there were differences in the number of *Nosema* spores between workers from the control group and those from the fed group (Kruskal–Wallis: *H* = 295.968, d.f. = 7, *n* = 360, *p* < 0.001). However, no significant differences were found between the infected (Kruskal–Wallis: *H* = 295.968, d.f. = 7, *n* = 360, *p* = 1.000) or control (Kruskal–Wallis: *H* = 295.968, d.f. = 7, *n* = 360, *p* = 1.000) workers from different colonies.

The lifespan of bees in the cages differed between individuals from different colonies (Kaplan–Meier survival analysis *χ*² = 14.27, d.f. = 3, *p* = 0.003; figure 2) and groups (Kaplan–Meier survival analysis = *Z* = 8.21, d.f. = 1, *p* < 0.001; figure 2). Infected individuals had a shorter lifespan compared to control bees.

The results of comparing PER reactions to different odours in the judgement bias test indicated no significant differences between bees from different colonies (general linear mixed model with repeated measurement: d.f. = 3; *F* = 0.270; *p* = 0.847), and therefore, data from different colonies were combined. In contrast, the analysis comparing PER reactions between workers from the control and infected groups revealed significant differences (d.f. = 1; *F* = 15.252; *p* < 0.001). Specifically, infected workers were more likely to extend their mouthparts in response to the three novel odours compared to control workers (7 : 3—d.f. = 1; *F* = 13.779; *p* < 0.001; 1 : 1—d.f. = 1; *F* = 8.092; *p* = 0.005; 3 : 7—d.f. = 1; *F* = 10.765; *p* = 0.001; figure 3b). However, there were no statistically significant differences between control and infected workers when exposed to the odours used during the conditioning phase (9 : 1—d.f. = 1; *F* = 1.034; *p* = 0.310; 1 : 9—d.f. = 1; *F* = 0.967; *p* = 0.326; figure 3c).

### (c) Experiment 3

First, we assessed the number of ovarioles between two groups of workers—control workers and more reproductively active rebel workers. To achieve this, we compared the number of ovarioles in the ovaries of these two groups across four experimental colonies. The results showed that rebel workers had a statistically greater number of ovarioles compared to control workers (Wald statistic = 54.04, *p* < 0.001; figure 4b). However, there were no statistical differences in the number of ovarioles among workers from different colonies (Wald statistic = 2.09, *p* = 0.554).

The lifespan of bees in the cages differed between individuals from different colonies (Kaplan–Meier survival analysis, *χ*² = 24.12, d.f. = 3, *p* < 0.002; figure 2, and groups (Kaplan–Meier survival analysis, *Z*= −8.89, d.f. = 1, *p* < 0.001; figure 2). Rebel workers had a longer lifespan compared to control bees.

The results of comparing PER reactions to different odours in the judgement bias test indicated no significant differences between bees from different colonies (general linear mixed model with repeated measurement: d.f. = 3; *F* = 1.000; *p* = 0.500), and therefore, data from different colonies were combined. In contrast, the analysis comparing PER reactions between workers from the control and rebel workers groups revealed significant differences (d.f. = 1; *F* = 26.482; *p* < 0.001). Specifically, control workers were more likely to extend their mouthparts in response to the three novel odours compared to rebel workers (7 : 3—d.f. = 1; *F* = 19.158; *p* < 0.001; 1 : 1—d.f. = 1; *F* = 19.062; *p* < 0.001; 3 : 7—d.f. = 1; *F* = 18.774; *p* < 0.001; figure 3b). However, there were no statistically significant differences between control and rebel workers when exposed to the odours used during the conditioning phase (9 : 1—d.f. = 1; *F* = 1.720; *p* = 0.191; 1 : 9—d.f. = 1; *F* = 0.855; *p* = 0.356; figure 3c).

## 4. Discussion

This study highlights the intricate relationships between expected longevity and cognitive biases in honeybee workers (*Apis mellifera*). The findings demonstrate that honeybees’ life expectancy is a pivotal factor influencing their behaviour and decision-making processes. Specifically, honeybees facing physical stressors due to injury, anaesthetic intervention [21], or infection [19,39] displayed a notable increase in optimistic judgement biases when confronted with ambiguous stimuli. This behavioural shift, as evidenced in experiments 1 and 2, suggests that bees with a reduction in lifespan exhibit a more risk-prone foraging strategy, characterized by an increased PER to novel odours. These results are consistent with previous research indicating that animals with shorter life expectancies often engage in riskier foraging behaviours [19,22].

Moreover, experiment 3 revealed that honeybee workers projected to have longer life spans [31] tend to adopt a more pessimistic cognitive bias. This implies that the perceived stability of their existence influences their resource search strategy, encouraging a more cautious and patient approach. The parallelism observed in cognitive biases among both shortened-longevity and longer-longevity bees underscores a broader ecological perspective, where life history and emotional states interweave to dictate survival strategies.

The relationship between expected longevity and performing tasks associated with varying levels of risk is compelling. Workers with shorter life expectancies seem to have a higher tendency to undertake tasks despite the associated risks, exemplifying the ‘division of labour by division of risk’ hypothesis [40]. Generally, eusocial insect workers undertake risky outside-nest tasks later in their life. Only two exceptions to this rule have been described, in a species of termite (*Zootermopsis angusticollis*) and in a species of ant (*Amblyopone pallipes*) [41]. Furthermore, workers who are either older, infected or have shorter life expectancies tend to perform other risky tasks as well. For instance, in leaf-cutting ants (*Atta cephalotes*), older workers often reside in the garbage chamber, taking on the responsibility of managing waste from younger transporting workers [42]. Similarly, in honeybees, foragers with shorter life expectancies may prefer to collect pollen—a task deemed riskier than nectar foraging [31,43]. These foragers also engage in more aggressive behaviours, such as stealing honey from other colonies [23], and may venture out to forage during unfavourable weather conditions, which further exemplifies their willingness to accept risks [40]. The propensity of workers with shorter life expectancies to undertake these risky tasks later in life enhances the overall fitness of the colony by extending the average worker lifespan [44,45]. This connection to heightened motivation, as illustrated in our study, underscores the adaptive strategies that these insects employ to optimize resource acquisition and contribute to colony survival.

It is particularly intriguing to consider how honeybees undergo physiological changes that are linked to a reduced life expectancy. Their physiology naturally shifts throughout their lifespan, influencing their behaviour in an automatic and instinctive manner. Many physiological alterations occur that are more closely related to the underlying biological signals of ageing and lifespan rather than to their actual age. These changes are also tightly connected to the division of labour within the colony. For instance, the transition from performing nest-related tasks to foraging is often accompanied by decreases in vitellogenin levels and increases in juvenile hormones in the haemolymph [46]. Such shifts in haemolymph composition can lead to a significant reduction in hemocyte counts, impairing their immune response [47]. Additionally, evidence indicates that bees engaged in riskier activities, like foraging trips, experience a decline in stored lipid reserves in their abdomen [48,49]. These physiological adaptations happen automatically, providing bees with internal cues about their body condition that influence their behaviour and motivation without any conscious awareness of their changing life expectancy.

Our results also indicated that some workers from the control groups exhibited the same high levels of judgement bias as those from the treatment groups. This finding is quite understandable. The hypothesis we tested assumes that all workers with shorter life expectancies should be inclined to undertake risky tasks, regardless of their assigned group. In our experiments, we utilized forager workers of various ages and randomly assigned them to experimental groups (experiment 1: control, injured and anaesthetized; experiment 2: control and infected). This randomness meant that some individuals in the control group could have shorter life expectancies compared to those in the other treatment groups. This assumption was validated in our cage test, although it is worth noting that most control workers lived longer than those in the treatment groups (figure 2). Some of the control workers had shorter lifespans than the longest-living anaesthetized, injured or infected bees. Therefore, we believe that these control workers with shorter lifespans could have possessed the same levels of judgement bias as their counterparts in the treatment groups.

In our study, we chose to utilize the go/no-go paradigm to assess judgement bias. This method involves training animals to approach a specific location for a reward (e.g. food) and to avoid approaching another location that may contain no food, unpalatable food or a mildly aversive stimulus [36]. The go/no-go paradigm is widely used in research on insect cognition, including bees. However, its has certain limitations, as the results may not solely reflect cognitive biases but could also be influenced by other factors such as motivation, arousal or attention [2,36]. An alternative method for studying judgement errors in bees is the active choice judgement bias test, which generally provides a more direct assessment of emotional states. This method involves training animals to discriminate between cues signalling high reward (CS+) and low reward (CS–) [36]. During testing, their responses to intermediate cues are observed—either through an active choice task with positive reinforcement), where animals are rewarded for choosing the CS+ and responses to the CS– are negatively reinforced, prompting animals to approach the CS+ to avoid a negative stimulus [36]. Although this alternative has has been used in only one study on bumblebees [50], the results indicated that physically stressed bees (e.g. shaking or trapping) were less optimistic than control individuals. We decided to use the go/no-go paradigm because it is less time-consuming, allowing us to test a larger number of individuals, which is a significant advantage given the constraints of our study. While the active choice judgement bias test may offer a more nuanced understanding of emotional states, its longer testing duration limits the number of subjects that can be practically examined in our research context. Even if the results may primarily indicate motivation levels rather than purely emotional states, they still highlight important differences among individuals, especially those with different lifespans, underscoring how age or life stage can influence behavioural responses.

Previous studies have shown that after experiencing stressful situations, bees tend to perceive ambiguous stimuli more pessimistically than those not exposed to such stressors. For example, it has been demonstrated that after their cages were shaken, agitated bees were more likely to interpret ambiguous stimuli as indicative of punishment and responded less frequently than individuals from the control group [11,50,51]. Conversely, when bumblebees received an unexpected reward before testing, their motivation to engage in activities and forage increased, indicating a positive emotional state [52]. At first glance, it might seem that our findings contradict those of the aforementioned studies, particularly regarding the effects observed in shaken bees. In our experimental setup, workers subjected to stressful treatments (injured, anaesthetized and infected) reacted more frequently to ambiguous stimuli compared to the control workers (figure 3). However, we can explain these differences in outcomes. In previous studies, animals were first trained to recognize specific signals before being subjected to stressors or unexpected rewards. Their behaviour was then tested, revealing that reactions and behaviours were directly influenced by the emotions elicited by their recent experiences prior to the test.

In our study, in experiments 1 and 2, the behaviour of bees was tested the day after the lifespan manipulations, whereas in experiment 3, where lifespan was determined during the larval stage, behaviour was assessed even after several days. Therefore, our focus was more on the long-term effects on life expectancy seem to overshadow the potential immediate impact of stressors. Initially, we exposed the bees to stress by shortening their life expectancies and subsequently trained them to respond to specific signals, followed by the judgement bias test. For this reason, the long-term effects of perceived life expectancy seem to overshadow the immediate emotional responses induced by stressors and reflect a more ingrained, adaptive behaviour towards resource acquisition under existential threat.

In conclusion, the findings from this study contribute to a broader understanding of how honeybees navigate the complexities of survival within their social structure. The interplay between cognition, motivation and life expectancy offers valuable insights into the behavioural ecology of these remarkable insects, pointing towards adaptive strategies that evolve in response to varying life histories. Future research should aim to unravel the mechanisms underlying these phenomena, particularly focusing on the implications for resource allocation and foraging behaviours in changing environments.

## Data Availability

Raw data are available on Dryad [53].
